# Sequential psychological and pharmacological therapies for comorbid and primary insomnia: study protocol for a randomized controlled trial

**DOI:** 10.1186/s13063-016-1242-3

**Published:** 2016-03-03

**Authors:** Charles M. Morin, Jack D. Edinger, Andrew D. Krystal, Daniel J. Buysse, Simon Beaulieu-Bonneau, Hans Ivers

**Affiliations:** Université Laval, École de psychologie, 2325 rue des Bibliothèques, Québec, QC G1V 0A6 Canada; National Jewish Health, 1400 Jackson Street, Denver, CO 80206 USA; Department of Psychiatry and Behavioral Sciences, Duke University School of Medicine, Box 3309, Durham, NC 27710 USA; Department of Psychiatry, University of Pittsburgh School of Medicine, 3811 O’Hara St., Pittsburgh, PA 15213 USA

**Keywords:** Insomnia, treatment, cognitive behavioral therapy, medication, combined therapy, sleep

## Abstract

**Background:**

Chronic insomnia is a prevalent disorder associated with significant psychosocial, health, and economic impacts. Cognitive behavioral therapies (CBTs) and benzodiazepine receptor agonist (BzRA) medications are the most widely supported therapeutic approaches for insomnia management. However, few investigations have directly compared their relative and combined benefits, and even fewer have tested the benefits of sequential treatment for those who do not respond to initial insomnia therapy. Moreover, insomnia treatment studies have been limited by small, highly screened study samples, fixed-dose, and fixed-agent pharmacotherapy strategies that do not represent usual clinical practices. This study will address these limitations.

**Methods/design:**

This is a two-site randomized controlled trial, which will enroll 224 adults who meet the criteria for a chronic insomnia disorder with or without comorbid psychiatric disorders. Prospective participants will complete clinical assessments and polysomnography and then will be randomly assigned to first-stage therapy involving either behavioral therapy (BT) or zolpidem. Treatment outcomes will be assessed after 6 weeks, and treatment remitters will be followed for the next 12 months on maintenance therapy. Those not achieving remission will be offered randomization to a second, 6-week treatment, again involving either pharmacotherapy (zolpidem or trazodone) or psychological therapy (BT or cognitive therapy (CT)). All participants will be re-evaluated 12 weeks after the protocol initiation and at 3-, 6-, 9-, and 12-month follow-ups. Insomnia remission, defined categorically as a score < 8 on the Insomnia Severity Index, a patient-reported outcome, will serve as the primary endpoint for treatment comparisons. Secondary outcomes will include sleep parameters derived from daily sleep diaries and from polysomnography, subjective measures of fatigue, mood, quality of life, and functional impairments; and measures of adverse events; dropout rates; and treatment acceptability. Centrally trained therapists will administer therapies according to manualized, albeit flexible, treatment algorithms.

**Discussion:**

This clinical trial will provide new information about optimal treatment sequencing and will have direct implication for the development of clinical guidelines for managing chronic insomnia with and without comorbid psychiatric conditions.

**Trial Registration:**

ClinicalTrials.gov Identifier: NCT01651442, Protocol version 4, 20 April 2011, registered 26 June 2012

## Background

Insomnia is characterized by difficulties initiating or maintaining sleep and associated with significant distress or impairments of daytime functioning that occur despite adequate opportunities for sleep [1–4]. More than 33 % of adults experience insomnia at least intermittently, whereas 10 % to 12 % suffer chronic sleep difficulties [5–7]. Although its significance is often minimized, persistent insomnia increases the risks for medical disorders, accidents, alcohol/drug abuse, and psychiatric illnesses [8–10]. When insomnia occurs co-morbid to a psychiatric illness such as major depression, it complicates disease management and often remains as a residual symptom that enhances risk for both suicide and relapse [11–13]. Moreover, insomnia contributes to increased healthcare utilization and costs [14]. More than 90 % of insomnia-related costs are attributable to work absences and reduced productivity [15].

### What are the treatment options?

Treatment options for insomnia include different classes of medications, psychological/behavioral therapies, and a variety of complementary and alternative therapies. The most widely used pharmacological agents are allosteric modulators of the GABA_A_ receptor complex, often referred to as benzodiazepine receptor agonists (BzRAs). These agents enhance the sleep-promoting effects of homeostatic sleep-drive and decrease activity in arousal systems (for example, acetylcholine, histamine, orexin/hypocretin, and serotonin) [16]. BzRAs include several benzodiazepines (for example, temazepam) and newer non-benzodiazepine agents (for example, zolpidem). Additionally, the melatonin receptor agonist, ramelteon; the orexin receptor antagonist, suvorexant; and the tricyclic, doxepin also have FDA approval for insomnia therapy. Finally, the use of antihistamines and the off-label use of sedating antidepressants such as trazodone and amitriptyline [17] are common for insomnia management. These agents enhance sleep by diminishing arousal through blocking the effects of wake-promoting systems [16].

Psychological/behavioral therapies target mechanisms thought to perpetuate insomnia such as maladaptive sleep habits, dysfunctional beliefs about sleep, excessive arousal, and poor sleep hygiene practices [18, 19]. Included among these approaches are various stand-alone strategies such as stimulus control, sleep restriction, relaxation training, sleep hygiene education, and cognitive therapy. Multicomponent, cognitive behavioral therapies (CBTs) that combine several of these therapeutic components to optimize outcomes have become the most frequently used approaches.

### How effective are the available insomnia therapies?

Of the various medications used for insomnia treatment, FDA-approved BzRAs have the most efficacy and safety data. Several meta-analyses, systematic reviews, and consensus statements have summarized the efficacy of drug therapies for insomnia [20–22]. For example, one meta-analysis [21] examined 22 placebo-controlled trials involving benzodiazepines and zolpidem in primary insomnia (PI) patients. This meta-analysis showed these agents produce reliable short-term (median treatment duration = 7 days; range = 4 to 35 days) improvements of sleep-onset latency (mean effect size = 0.56), number of awakenings (effect size = 0.65), total sleep time (effect size = 0.71), and sleep quality (effect size = 0.62). A few long-term trials [23–25] have shown that BzRAs such as zolpidem and eszopiclone have continued efficacy and safety for periods of 3 to 12 months of nightly use. Most treatment studies have focused on primary insomnia, that is, insomnia not due to another medical, psychiatric, or sleep disorder. Whereas studies of BzRAs with insomnia occurring comorbid to a mental disorder have been limited, some data have shown that combining a BzRA with antidepressant medications (SSRIs) is more efficacious than SSRIs alone for treating both insomnia and depression in patients with major depressive disorder [26–28]. In addition, trazodone is used widely “off-label” for insomnia treatment [17] and some data support its efficacy for treating insomnia occurring comorbidly with major depression [29–33].

Psychological and behavioral therapies for insomnia are also well supported by the scientific evidence. Meta-analyses and systematic reviews [22, 27, 34, 35] indicate that such treatments produce moderate to large improvements in sleep onset latency (effect sizes = .87 to .88), total sleep time (effect sizes = 0.42 to 0.49), number of awakenings (effect sizes = 0.53 to 0.63), duration of awakenings (effect size = 0.65), and sleep quality ratings (effect size = 0.94). Approximately 70 % to 80 % of patients benefit from treatment, with the best outcomes resulting from multifaceted therapies such as cognitive behavioral therapy (CBT) [19, 35]. Most of the evidence is derived from studies of patients with primary insomnia. Applications of these therapies to patients with insomnia and comorbid psychiatric or medical disorders have been more limited, but the available data suggest these treatments produce sleep improvements among patients with chronic pain [36], breast cancer [37], fibromyalgia [38], mixed medical disorders [39], and depression [40, 41]. These therapies also lead to improvements in mood status and reductions in other disease-specific symptoms [37, 38, 40].

### What should be our first stage therapy, and what should we do when that fails?

Deciding whether to use pharmacological or psychological/behavioral therapy is difficult because both forms of treatment have their advantages and limitations [42, 43]. Medications usually produce rapid improvements, are widely available, and generally well tolerated, but adverse effects (for example, daytime sedation) and a risk of tolerance and dependence may complicate their use. Furthermore, no data document the enduring benefits of these agents after their discontinuation. In contrast, psychological/behavioral therapy has minimal side effects, is preferred by many patients, and results in enduring sleep improvements long after termination of treatment [43]. However, these therapies require more extensive provider contact and have a slower therapeutic action than medications. In addition, they are less widely available than medications despite recent efforts to facilitate their implementation through abbreviated therapy protocols [44], self-help interventions [45], and Internet-based programs [46, 47].

Few head-to-head comparisons of medications and psychological/ behavioral therapies [48–51] have been made. In general, studies that compared BzRA therapy, CBT, and combined BzRA/CBT therapy showed little difference in short-term outcomes, but superior longer-term outcomes with CBT, compared to BzRA and combined treatment. In contrast, a sequential treatment strategy that commenced with 6 weeks of combined CBT/BzRA therapy followed by an extended 6 months of CBT alone proved superior to the continued long-term combined therapy or CBT provided in the absence of any medication [52]. Although informative, these studies are limited by their small sample sizes, use of fixed-dose/fixed-agent pharmacotherapy strategies that do not represent standard clinical practice, and their predominant focus on patients with primary insomnia. Hence, these findings provide limited guidance for decisions concerning the optimal first-stage insomnia therapy and for the more challenging subgroup of patients with comorbid psychiatric illnesses. Some evidence suggests that patients with comorbidities have more difficulties complying with behavioral interventions [40], and they may not respond as well to treatment, regardless of its specific modality, as do patients with primary patients [53]. In addition, a large proportion of patients fail to remit with first-stage therapy and retain residual insomnia symptoms. In such situations, switching from one therapy to another, on a trial and error basis, is common clinical practice. However, no studies have examined which first-stage treatment is optimal for both primary and comorbid insomnias and which second-stage treatment offers the best “added value” for patients who do not remit following psychological or medication first-stage therapy.

### Objectives

The main objectives of this study are to compare short- and long-term outcomes of psychological (behavioral and cognitive) and pharmacological therapies (zolpidem and trazodone) used sequentially for insomnia among patients with and without comorbid psychiatric disorders. Specifically, the study will compare the efficacy of Behavior Therapy (BT) and zolpidem (ZOL) as first-stage therapies and examine the moderating effect of psychiatric comorbidity on outcomes. For those who do not remit with the first-stage therapy, we will evaluate the added value of Cognitive Therapy (CT) and trazodone (TRA) as second-stage therapy.

## Methods

### Study design and setting

**Fig. 1:**
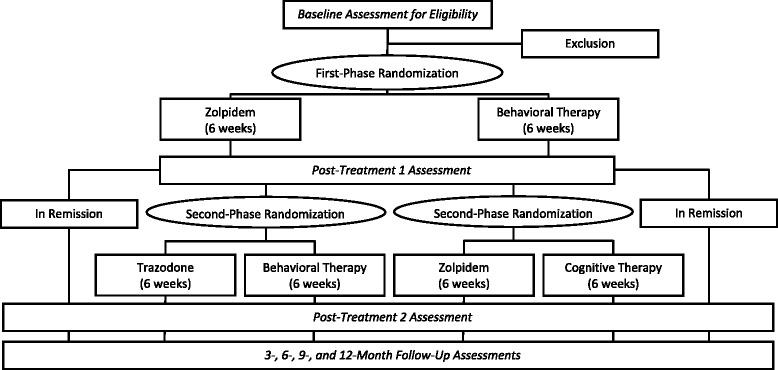
Flow chart of study design

The study is a randomized controlled trial with two treatment stages and two treatment modalities (psychological therapy – behavior therapy and cognitive therapy; medication – zolpidem and trazodone) for each stage (see Fig. 1 for study design flow chart). Participants meeting criteria (*N* = 224) are randomly assigned in a 1:1 ratio to zolpidem therapy (ZOL; *n* = 112) or behavioral therapy (BT; *n* = 112), stratified by gender, age (<55 years versus ≥ 55 years), and presence or absence of a comorbid psychiatric disorder. Randomization is based on a computer-generated list of numbers for each site and the participants’ allocation is concealed by using sequentially numbered sealed-envelopes that are opened by study coordinators only after a patient meets all selection criteria and is ready to initiate treatment. Patients will be randomly assigned to first-stage treatment involving either zolpidem or behavioral therapy (BT). After this initial 6-week therapy, patients in remission will be followed for the next 12 months on maintenance therapy while nonremitters will be re-randomized to a second-stage treatment involving pharmacotherapy (zolpidem or trazodone) or psychological therapy (BT or cognitive therapy (CT)). All participants will be re-evaluated after second-stage therapy, and at 3-, 6-, 9-, and 12-month follow-ups. Insomnia remission, defined as a score of less than 8 on the Insomnia Severity Index, will serve as the primary outcome. Key secondary outcomes will include sleep variables from diary and PSG measures; ratings of sleep quality and daytime functions; adverse events; and treatment acceptability.

Two sites are involved in the recruitment of patients: Université Laval, Québec City, Quebec, Canada, and the National Jewish Health, Denver, Colorado, United States. A third site, Duke University, is involved in analyzing polysomnographic (PSG) data. The study protocol has been approved by the ethics committees at the two main sites: the Research Ethics Board, Institut universitaire en santé mentale de Québec, Québec, Canada, and the Institutional Review Board (IRB) of National Jewish Health, Denver, Colorado, USA. All important protocol modifications will be communicated to both local IRBs. Study coordinators obtain written informed consent from all prospective participants at their first screening visit.

### Participants

A total of 224 adults (aged 21 and older) with chronic insomnia are recruited from the community and from outpatient medical and mental health clinics and through media advertisements, flyers, and letters to primary care physicians and mental health clinicians. The inclusion and exclusion criteria are presented in Table 1. These criteria are broad enough to obtain results widely generalizable to the insomnia patient population commonly seen in clinical practice, including those with primary insomnia and insomnia comorbid to a psychiatric disorder. Individuals with a comorbid medical condition are excluded only if the medical condition is life threatening or would contraindicate the use of study medications.

**Table 1:** Selection criteria

Inclusion criteria (combination of criteria from the Diagnostic and Statistical Manual of Mental Disorders, 4th edition (DSM-IV), Insomnia Research Diagnostic Criteria, and International Classification of Sleep Disorders, 2nd edition
▪ Males and females age ≥ 21 years
▪ Complaint of persistent (that is, > 1 month) difficulties initiating or maintaining sleep despite adequate opportunity for sleep
▪ Sleep onset latency or wake time after sleep onset ≥ 30 minutes for three or more nights per week during 2 weeks of sleep diary monitoring
▪ Insomnia Severity Index (ISI) total score > 10, indicating at least “mild” insomnia
▪ Score ≥ 2 on either the interference or distress item of the screening ISI, indicating the insomnia causes significant distress or impairment in social, occupational, or other areas of functioning
Exclusion criteria
▪ Untreated psychiatric disorder (for example, major depression), as these conditions have specific treatments and it would be inappropriate not to offer those treatments
▪ Lifetime diagnosis of any psychotic or bipolar disorder, as sleep restriction and medications for insomnia may precipitate mania and hallucinations
▪ Imminent risk for suicide
▪ Alcohol or drug abuse within the past year
▪ Terminal or progressive physical illness (for example, cancer or COPD) or neurological degenerative disease (for example, dementia)
▪ Current use of medications known to cause insomnia (for example, steroids)
▪ Sleep apnea (apnea/hypopnea index > 15), restless legs syndrome, periodic limb movement during sleep (PLMS with arousal > 15 per hour), or a circadian rhythm sleep disorder (for example, advanced sleep phase syndrome)
▪ Personal or familial (first-degree relatives) history of sleepwalking
▪ Women being pregnant or expecting to become pregnant during treatment

Individuals using sleep-promoting medications (prescribed or over-the-counter) are included if they are willing and able to discontinue medications at least 2 weeks before the baseline assessment. Participants using alcohol as a sleep aid or alcohol after 7:00 pm on a regular basis are required to discontinue this practice at least 2 weeks prior to the baseline assessment. Individuals using psychotropic medications (for example, anxiolytics and antidepressants) are not automatically excluded from the study. Those on stable dosages (for at least 3 months) of SSRI or SNRI medications, and who show at least partial remission from their mood or anxiety disorder, are accepted in the study if they meet the selection criteria above. Patients using TCAs, MAOIs, or atypical antidepressants are excluded even if in remission, as the effects of these medications on sleep might confound interpretation of the findings.

### Measures

#### Screening instruments

Structured clinical interviews are used to screen potential participants. The Insomnia Interview Schedule [54] is an interview assessing the nature, severity, and frequency of the current insomnia problem, as well as its history, course, and contributing factors. The Duke Structured Interview for Sleep Disorders [55] is used to assist in ascertaining sleep disorder diagnosis according to DSM-IV-TR [56] and International Classification of Sleep Disorders criteria [3]. This instrument has acceptable reliability and discriminant validity [57]. In addition, the Structured Clinical Interview for DSM-IV Axis I Disorders (SCID) [58] is used to classify enrollees as having primary or comorbid insomnia and to identify study candidates with disorders leading to exclusion. All interviews are audiotaped and reliability checks are conducted on 15 % of them. The Folstein Mini-Mental State Exam (MMSE) [59] is administered to exclude participants with cognitive deficits (MMSE score < 27) that make them unable to give informed consent or fully participate in an interactive treatment.

#### Sleep diary

Subjective estimates of sleep and wake times are obtained daily using a web-based sleep diary. Participants are instructed to report the following information about their previous night’s sleep: bedtime, sleep onset latency, number and length of nocturnal awakenings, time of final waking, rising time, and subjective rating of sleep quality. Additional questions query daytime napping, and caffeine, alcohol, and sleep medication use. Participants with missing data are contacted by phone to remind them to fill out the diary daily. Electronic sleep diaries are obtained for 2 weeks at baseline and at each subsequent assessment period, as well as for the entire treatment period (first- and second-stage).

#### Polysomnography

Participants undergo ambulatory nocturnal polysomnographic (PSG) monitoring at their home using their typical bedtimes and arising times for baseline/screening (two nights) and outcome assessment (2 nights after each treatment phase). PSG monitoring is conducted according to standard procedures with regard to montage and sampling rate (256 Hz) and records are scored, blind to treatment assignment, in 30-second epochs using standard criteria [60] for sleep staging and sleep-associated events (for example, apneas). Scoring is done at the Duke University Insomnia and Sleep Research Program by trained technologists under the supervision of an experienced polysomnographer who is board-certified in sleep medicine (AK).

#### Outcome measures

The primary study end point is the proportion of individuals achieving remission, using total scores from the Insomnia Severity Index (ISI) [61, 62] completed during the last treatment visit and at the following week (weeks 5 and 6 of each treatment stage). *Remission* is defined as a mean ISI score < 8 for weeks 5 and 6, with neither one of these two ISI score > 10. The ISI is a seven-item self-report questionnaire that provides a global measure of perceived insomnia severity based on several indicators (for example, difficulty falling or staying asleep, satisfaction with sleep, and degree of impairment with daytime functioning). The total score ranges from 0 to 28: 0 to 7 (no clinical insomnia), 8 to 14 (subthreshold insomnia), 15 to 21 (insomnia of moderate severity), and 22 to 28 (severe insomnia). The ISI has been validated extensively and has proven sensitive to therapeutic changes [52, 62].

Several secondary outcomes are also monitored including sleep/wake variables: sleep onset latency (SOL), wake time after sleep onset (WASO), total sleep time (TST), sleep efficiency (SE) derived from both sleep diaries and PSG. Participants also complete the Pittsburgh Sleep Quality Index [63] and the Dysfunctional Beliefs and Attitudes about Sleep scale [54] at each assessment to examine changes in overall sleep quality and sleep-related cognitions. A clinical global improvement rating is completed by a blinded rater after each treatment. This rater also interviews participants at the conclusion of their study involvement to determine whether they continue to meet diagnostic criteria for insomnia.

To examine treatment-related changes in daytime functions, participants complete the Multidimensional Fatigue Inventory (MFI) [64], the SF-36 Health Survey (SF-36) [65], and Work and Social Adjustment Scale (WSAS) [66]. The MFI is a 20-item scale [64] assessing several dimensions of fatigue (for example, physical and mental). The SF-36 is a quality-of-life measure that comprises eight scales and two summary measures (physical health and mental health) [65]. The WSAS assesses the functional impact of a specific disorder (in our study, insomnia) on five domains: ability to work, home management, social leisure activities, private leisure activities, and relationships. Changes in mood status are assessed with the Beck Depression Inventory-II (BDI-II) [67] and the Trait part of the State-Trait Anxiety Inventory (STAI-Trait) [68].

Adverse events (AE) are monitored with the Systematic Assessment for Treatment Emergent Events (SAFTEE), a reliable and valid instrument for assessing AEs related to study treatments [69]. AEs are also monitored for patients treated with BT and CT because some interventions (for example, sleep restriction) may produce excessive daytime somnolence. Finally, we use an amended version of the Therapy Evaluation Questionnaire to assess treatment credibility, acceptability, and patient satisfaction [70].

### Procedures

After an initial telephone screening for eligibility, study candidates complete several paper-and-pencil screening measures (see Table 2 for details). Then, they complete PSG to rule out other sleep disorders (for example, apnea and PLMS). In the absence of such disorders during the first PSG screening night, a second consecutive PSG is conducted for baseline assessment. After completing baseline assessment, patients meeting criteria are randomized to one of the first-stage therapies. After completing the initial 6-week treatment, remitters remain on maintenance therapy. Remitters from the zolpidem condition attend a monthly follow-up visit with the physician, who administers the SAFTEE and provides medication supply for nightly use (if needed) until the next visit. Remitters from the BT condition receive monthly phone calls during which the SAFTEE is administered, but there are no further therapy visits. Nonremitters following first-stage treatment are encouraged to accept randomization to a second-stage treatment provided over the next 6 weeks. Nonremitting participants treated with BT initially are randomized either to another psychological treatment, that is, cognitive therapy (CT), or to a medication therapy (ZOL). Those treated with medication (ZOL) initially are tapered off their medication and randomized to BT or to a different medication (trazodone, TRA). During this second treatment, all participants continue to complete a nightly sleep diary, the ISI (weekly), and other questionnaires (at week 12), whether they receive a second therapy or not. Measurements are taken at baseline, at the end of first- and second-stage therapies (that is, weeks 6 and 12), and at follow-ups conducted 3, 6, 9, and 12 months after the week 12 assessment.

**Table 2:** Timing of clinical assessments and measures throughout the study

Instruments/time point	Screen	Baseline	Tx-1	Post-1	Tx-2	Post-2	3-month FU	6-month FU	9-month FU	12-month FU
Medical history/physical exam	X									
Mini-Mental State	X									
Vital signs	X			X		X	X	X	X	X
Insomnia Interview Schedule	X			X		X	X	X	X	X
Duke Structured Interview for Sleep Disorders	X			X		X	X	X	X	X
Structured Clinical Interview for DSM-IV Disorders	X							X		X
Sleep diary	X	X	X	X	X	X	X	X	X	X
Polysomnography	X ^a^	X ^a^		X		X				
Insomnia Severity Index	X	X	X	X	X	X	X	X	X	X
Clinical Global Improvement				X		X	X	X	X	X
Pittsburgh Sleep Quality Index		X		X		X	X	X	X	X
Dysfunctional Beliefs and Attitudes about Sleep Scale		X		X		X	X	X	X	X
Multidimensional Fatigue Inventory		X		X		X	X	X	X	X
SF-36 Health Survey		X		X		X	X	X	X	X
Work and Social Adjustment Scale		X		X		X	X	X	X	X
Beck Depression Inventory		X		X		X	X	X	X	X
State-Trait Anxiety Inventory		X		X		X	X	X	X	X
Systematic Assessment for Treatment Emergent Events		X	X	X	X	X	X	X	X	X
Treatment Evaluation Questionnaire			X	X	X	X		X		X

FU, follow-up; Tx-1, first-stage treatment 1; Tx-2, second-stage treatment

^a^ Screening (full montage to rule out other sleep disorders) and baseline (sleep montage only) assessments are conducted on consecutive nights

Participants receiving second-stage therapy complete two additional PSG nights at week 12. Subsequently, all participants enter follow-up and are contacted monthly for adverse event monitoring (that is, in-person visits with the physician for participants receiving medication in second-stage therapy; phone calls by the research coordinator for participants in the BT and CT conditions). For all participants, in-person visits occur 3, 6, 9 and 12 months after the end of the second-stage treatment, when participants are interviewed with the Duke Sleep Interview Schedule to determine if they meet criteria for insomnia disorder. They are asked to complete additional electronic sleep diaries (2 weeks), outcome questionnaires, and the SAFTEE. SCID interviews serve to document psychiatric diagnoses at the 6- and 12-month follow-ups.

### Treatments

#### Psychological therapy

The first-stage psychological therapy consists of behavioral therapy (BT), which includes sleep restriction [71] and stimulus control procedures [72]. These well-established strategies are designed to strengthen homeostatic sleep drive, consolidate sleep via reducing time in bed, establish a regular sleep schedule, and curtail sleep-incompatible behaviors. Although full CBT may represent the psychological treatment of choice, we have chosen to employ only its core behavioral elements as first-stage therapy because it is briefer, easier to deliver, and, ultimately, more transferable to various clinical settings.

The second-stage psychological treatment consists of cognitive therapy (CT). CT is aimed at altering sleep-disruptive and mood-disturbing cognitions that exacerbate the vicious cycle of insomnia. Such cognitions are typically related to thoughts and beliefs about unmet sleep requirements and potential insomnia consequences. Perpetuating mechanisms such as excessive self-monitoring and worries are also prime targets for CT. CT follows standard procedures to identify and alter these sleep-interfering cognitions via recording automatic thoughts, Socratic questioning, constructive worry, and behavioral experiments [18, 73].

We chose CT as a second-stage therapy because it is more time consuming, requires more training, and may not be essential for all patients with insomnia. Yet, because of its unique features in targeting some critical cognitive perpetuating mechanisms (for example, worries) shared by insomnia and some comorbid psychiatric disorders (for example, anxiety, and depression) [74], and not specifically addressed by BT, the addition of CT as a second-stage therapy provides an opportunity to evaluate its unique contribution to outcomes among patients with comorbid psychiatric disorders. This condition is also likely to make the switch within the psychological treatment modality (that is, from BT to CT) more equivalent conceptually to the switch within the pharmacotherapy modality (that is, from zolpidem to trazodone).

#### Medication

The first-stage pharmacological treatment involves zolpidem (ZOL), sublingual, 5 to 10 mg, taken nightly at bedtime. The choice of zolpidem as a first-stage therapy was based on the extensive literature documenting its efficacy for insomnia and on the fact that it is among the most commonly prescribed medications for insomnia [29, 33, 75]. It has also been shown efficacious for insomnia in depressed patients treated with antidepressant medications [76]. All participants start with an initial dose of 5 mg, which is titrated up to 10 mg based on therapeutic response, side effects, and the patient’s age and gender (based on FDA recommendations, dosage is limited to 5 mg in women). Support and encouragement to comply with the prescribed medication regimen is provided by the prescribing physician, but no behavioral or cognitive intervention is allowed during these consultations visits. All medications are dispensed by the pharmacy at each site.

The second-stage pharmacotherapy consists of trazodone (TRA; 50 to 150 mg), taken 30 minutes before bedtime. We chose trazodone because it is also among the most commonly prescribed medications for insomnia (off-label) in clinical practice [29, 33]. It has a different mechanism of action than BzRAs and has shown efficacy for insomnia co-occurring with major depression [30–32, 77].

At the end of the first-stage treatment, participants who do not achieve remission on medication receive a final drug supply and a written withdrawal schedule (designed by the study physician). They are informed of possible rebound insomnia and instructed not to discontinue medication abruptly. Although the time required to discontinue medication may vary across individuals, a 2- to 3-week taper schedule is adequate for most. Those who achieve remission with medication provided as a first-stage treatment, as well as those provided a medication for second-stage treatment, stay on medication through follow-ups. Subsequently, these patients use a similar discontinuation schedule at the end of the 12-month follow-up. Those who wish to continue medication are referred to their primary care physician for further follow-ups.

### Treatment implementation and monitoring

All four treatments are administered in the context of four, individual, sessions led by clinical psychologists (BT, CT) or physicians/physicians’ assistants (ZOL, TRA) spread over a 6-week period. Consultation visits take place at weeks 1, 2, 4, and 6 and last 50 minutes for psychological treatments and 20 minutes for medication treatments. In addition to the main content pertaining to each treatment modality, both first-stage treatments (BT and ZOL) include generic sleep hygiene education about basic sleep hygiene education about the impact of stimulants, alcohol, caffeine, and exercise on sleep. The ISI and SAFTEE are administered at all consultation visits of both treatment stages. Clinicians use treatment manuals and receive ongoing supervision during the course of the study to standardize treatment administration. Therapy sessions are audiotaped and approximately 10 to 15 % of those sessions will be rated with a standard checklist by blinded raters for the presence of essential ingredients of a given treatment and for the absence of proscribed treatment instructions. Participants’ compliance with treatment protocols (for example, time spent in bed and the use of medication) are monitored by treating clinicians via sleep diaries and pill count. Treatment is discontinued or modified if adverse events are reported. During the trial, any treatment deemed necessary for other medical or psychiatric conditions is permitted.

### Data management and analysis

#### Data management

All case record forms are anonymized to ensure confidentiality. Electronic sleep diaries are used to foster adherence with daily data monitoring and minimize data collection errors. Data entry for other paper-and-pencil measures is completed at each site in an Access database by research assistants. A statistician revises data periodically to identify missing or incoherent data. Upon completion of the study, we will investigate missing data patterns to ascertain if data are missing completely at random, at random or not at random [78]. Our primary analyses will use statistical models robust to the first two data patterns, although we will perform sensitivity analyses for missing data not at random considering specific reasons for attrition if needed [79]. No data imputation will be performed, and all available observations will be included in inferential analyses. Trial results will be communicated through publications in peer-reviewed journals, scientific conferences, and lay public conferences.

#### Primary outcome analyses

All primary outcome analyses will be conducted based on the Intention-to-Treat principle. Accordingly, all patients with at least one set of post-baseline data will be included in these analyses. To control for possible site effects, the clinical site will be included as a main effect, and the inclusion of a clinical site interaction with other main effects (when appropriate) will be investigated for significance.

##### Hypothesis 1a

The proportion of patients achieving remission with the first-stage therapy and sustain remission through follow-up will be higher among those receiving BT than among those receiving zolpidem. A standard logistic regression will be used to compare the probability of short-term (after first-stage treatment) and sustained remission (primary outcomes) between BT and zolpidem conditions. Sustained remission is defined as remission being achieved after first-stage therapy and maintained at 12 weeks (and at the 12-month follow-up).

##### Hypothesis 1b

A lower proportion of CMI patients will achieve remission with first-stage therapies than will those with PI. A similar logistic regression will be used to compare the probability of short-term (after first-stage treatment) and sustained remission according to the presence or absence of psychiatric comorbidity. This effect will be investigated as a moderator of the impact of first-stage treatment on remission. The significance of a moderator is presumed by a significant moderator × treatment interaction. To investigate the moderating effect of psychiatric comorbidity, this variable and its interaction with treatment will be added as fixed effects to the model. A significant interaction will then be explored using simple effects to test if CMI is associated to poorer acute and long-term outcomes for each first-stage therapy.

##### Hypothesis 1c

Secondary outcomes (sleep, fatigue, and mood) will show greater improvements through treatment and follow-up for those receiving BT than for those receiving zolpidem. Group comparisons for secondary outcome measures will be performed using a mixed model approach [80], including a generalized mixed-effect regression models for binary variables (for example, diagnosis for insomnia) and a normal mixed-effect regression model for continuous variables. In both modeling approaches, treatment, time, and potential confounding factors will be included in the models as fixed main effects. The models will also include random effects for intercept and slope (patient by time) for each subject.

##### Hypothesis 2a

The insomnia remission rate after the second-stage treatment for all conditions combined will be 20 % higher than with first-stage treatment (that is, increment from 40 % to 60 %). Since participation in the second randomization is conditional upon not remitting with first-stage therapies, generalized estimating equations models (GEE) will be used to test whether the overall remission rate significantly increased.

##### Hypothesis 2b

Of all patients who enter second-stage treatment, a greater proportion who switches modalities (from psychological to medication treatment, or vice-versa) will achieve remission and sustain it through follow-up than will those staying within a treatment modality. To maximize degrees of freedom, weighted GEE models will be used to compare remission rates after 12 weeks of treatment and sustained remission after 12 months, according to two main effects: (a) having received BT or zolpidem during Stage I and, (b) having switched treatment modality or not for second-stage treatment. These main effects and their interactions will capture the partial effect of each treatment combination while accounting for relevant patient trajectory (data from first- and second-stage therapies). Other main fixed effects (for example, time) and potential baseline covariates will be included. Weights will be computed for each case after specifying a dropout model estimated from the observed dropout patterns.

##### Hypothesis 2c

CMI patients who enter second-stage treatment will show a higher remission rate with treatments that target sleep and mood symptoms (CT and trazodone) than with treatments targeting primarily sleep (BT and zolpidem). This added therapeutic effect will be higher in CMI than in PI patients. Two tests will be of primary interest within the full factorial (comorbidity × conditions × time) weighted GEE model: (a) an a priori contrast to examine remission rates after second-stage treatment according to whether CMI patients received the “mood addressing” sequences (BT → CT or ZOL → TRA) or the “non-mood” sequences (BT → ZOL or ZOL → BT), and (b) a comorbidity × treatment interaction will be used to compare whether the added therapeutic effect obtained by addressing mood symptoms is higher for CMI than PI patients. Significant interactions will be examined with simple effects to test whether temporal changes observed for each subgroup during second-stage therapies are significant. Other fixed effects such as first-stage therapy and baseline covariates will be included.

##### Hypothesis 2d

Secondary outcomes will show response patterns consistent with Hypotheses 2a through 2c. When the outcomes are assessed on a binary scale, identical analyses will be performed. When the outcome are assessed on a continuous scale, weighted GEE will be specified with a normal distribution and an identified link function.

#### Sample size and detectable effect size

All sensitivity power analyses were computed following procedures outlined by Stroup [81] for mixed models and Dahmen et al. [82] for weighted GEE models and were based on a two-tailed 5 % alpha and 80 % power. Based on studies conducted by our group [52, 83] and on recent reviews [84], attrition rates of 10 % were used as attrition estimates in the computation of detectable differences.

Power computations for the hypotheses related to the primary outcome (remission rate) are reported here using the GEESIZE program [82]. For hypothesis 1a, our expected sample size of 224 (*n* = 200 at post-1 after attrition) will give a standard 80 % power to detect a difference of 17.7 % in sustained remission rates between BT and zolpidem after stage-1 therapies. For hypothesis 2a, the initial sample size of 224 (effective sample at Post 2 = 178 after taking into account attrition) will give a standard power to detect a increment of 9.9 % in remission rates between Post I and Post II assessments (all conditions confounded), assuming a working correlation of 0.70. For hypothesis 2b, the same sample after attrition, excluding patients who are already remitted (effective sample size = 90) will give a standard level of power to detect a difference of 18.3 % in remission rates at Post II between patients who switched treatments and those who did not. If remitted patients are included to increase power, sensitivity would also be increased, allowing the detection of a difference of 13.3 % or larger in remission rates between patients who switched treatments and those who did not.

## Discussion

This study addresses research priorities set forth in the 2005 NIH State-of-the-Science Conference on the Manifestations and Management of Chronic Insomnia in Adults. As noted in the summary statement from that conference, “…little is known about the comparative benefits of these treatments, their combination, and their effects on understudied features of chronic insomnia. To address this lack of knowledge, randomized controlled trials will be required that are large scale and multi-site and compare at least two effective or promising treatments. This should include comparisons between pharmacological agents as well as between those agents and CBT.” This dual-site trial is specifically designed to address such objectives and to test the efficacy of such treatments in patients with both primary and comorbid forms of insomnia.

Currently, few data are available concerning the relative efficacy of the psychological/behavioral and BzRA therapies for managing chronic insomnia disorder since large multisite head-to-head comparisons of these treatments have yet to be conducted. Furthermore, there are virtually no data on how well each of these treatment approaches performs in producing and sustaining insomnia remission over time. Finally, the value of sequenced treatments for patients who fail to achieve insomnia remission when BT or BzRA are used as first-stage therapies has yet to be investigated. The current study was designed to address these gaps in the insomnia treatment literature. It has the following innovative features: 1) enrollment of participants with chronic insomnia disorder, with and without psychiatric comorbidity; 2) use of the clinically-relevant primary outcome, insomnia remission; 3) use of a sequential treatment design that tests various first-stage-to-second-stage treatment sequences; 4) flexible medication dosing, rather than a fixed-agent/fixed dose design; and 5) collection of AE data for both psychological and medication therapies so that the relative safety of the two approaches can be examined.

Given the high prevalence, morbidity, and societal costs of insomnia, this study is likely to yield new information that will have direct implications for the clinical management of insomnia, and as such, it will benefit both individuals who suffer from insomnia and society in general. This project should provide new and relevant information that contributes to the development of clinical guidelines for CMI and PI management, guidelines that are critically lacking.

### Trial status

The trial is currently in the active recruitment phase. As of 30 December 2015, 185 participants of the required 224 have been enrolled.

## Abbreviations

AE: adverse events
BT: behavioral therapy
BzRA: benzodiazepine-receptor agonists
CBT: cognitive behavioral therapy
CMI: comorbid insomnia
CT: cognitive therapy
GEE: generalized estimating equations models
IRB: institutional review board
ISI: insomnia severity index
MMSE: mini-mental status examination
PI: primary insomnia
PSG: polysomnography
SAFTEE: systematic assessment for treatment emergent events
SCID: Structured Clinical Interview for DSM-IV
TRA: trazodone
ZOL: zolpidem
